# 2719. Oral Antibiotics for Treatment of Gram-Negative Bacteremia in Solid Organ Transplant Recipients: A Propensity Score Weighted Retrospective Observational Study

**DOI:** 10.1093/ofid/ofad500.2330

**Published:** 2023-11-27

**Authors:** Eliezer Zachary Nussbaum, Sophia Koo, Camille N Kotton

**Affiliations:** Tufts Medical Center/Tufts University School of Medicine/Division of Geographic Medicine and Infectious Disease, Cambridge, Massachusetts; Brigham and Women's Hospital, Dana-Farber Cancer Institute, Boston, Massachusetts; Massachusetts General Hospital, Boston, Massachusetts

## Abstract

**Background:**

Treatment of gram-negative bloodstream infections (GNBI) with oral rather than intravenous (IV) antimicrobial agents can reduce length of stay, catheter associated complications and improve patient satisfaction. There is minimal data regarding this practice in solid organ transplant recipients (SOTR). We assessed the safety and efficacy of oral step-down therapy for treatment of uncomplicated GNBI in SOTR.

**Methods:**

We identified all SOTR within the Massachusetts General and Brigham and Women’s Hospital systems from 2016-2021 with uncomplicated GNBI that were transitioned from IV to oral agents to complete treatment. We identified a second group of patients with uncomplicated GNBI who remained on IV antimicrobials for the entirety of treatment but had an infecting organism susceptible to at least a moderately bioavailable oral agent. We conducted a propensity score weighted retrospective study to compare outcomes of the oral and IV groups. Primary endpoints were mortality, recurrence of bacteremia and re-initiation of IV antibiotics within 30 days of treatment completion. Secondary endpoints included length of stay, *C. difficile* infection, treatment associated complications, and need for tunneled central venous access.

**Results:**

120 GNBI from 107 patients met inclusion criteria in the oral group and 42 GNBI from 40 patients in the IV group. There was no statistically significant difference in mortality, bacteremia recurrence, or re-initiation of IV antibiotics between oral and IV groups. Compared with the IV group, patients transitioned to oral antibiotics had an average length of stay that was 1.97 days shorter (p=0.005, 95% CI -3.56, -0.39 days). Odds of developing *C. difficile* were 8.4 times higher in the IV group (p=0.015, 95% CI 1.5, 46.6) and the odds of developing other treatment associated complications were 6.4 times higher in the IV group (p=.002, 95% CI 1.9-20.9). 55% of patients in the IV group required placement of a tunneled central venous catheter. There was no difference in total duration of antibiotic treatment between groups.

Baseline Patient Characteristics

**Figure:**
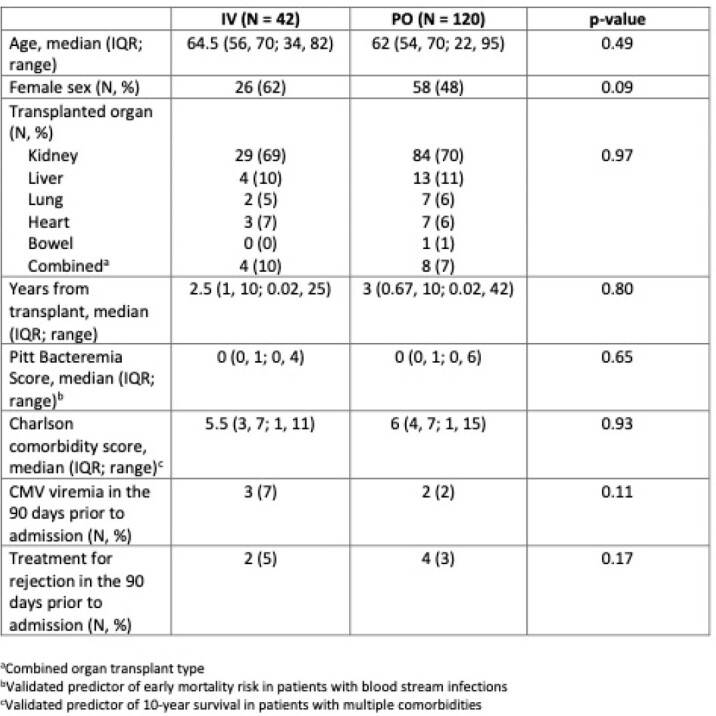

Infecting Species, Source of Infection and Antibiotic Therapy

**Figure:**
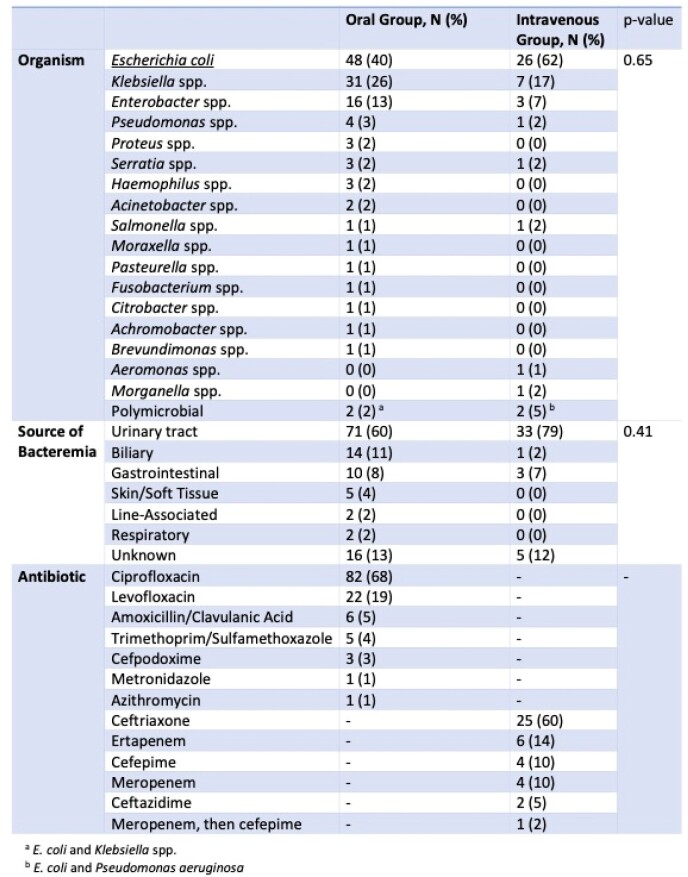

Clinical Outcomes in Oral and IV Antibiotic Treatment Groups

**Figure:**
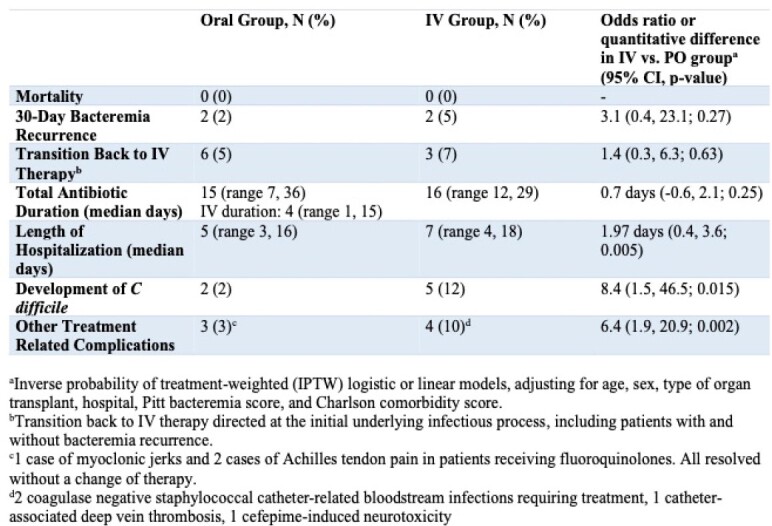

**Conclusion:**

Conversion from IV to oral therapy with moderate to highly bioavailable antimicrobials was effective and associated with fewer treatment related adverse events in SOTR with uncomplicated GNBI.

**Disclosures:**

**Sophia Koo, MD, SM**, Aerium Therapeutics: Advisor/Consultant|GSK: Grant/Research Support|Merck: Grant/Research Support|Scynexis: Grant/Research Support

